# RNA-seq analysis reveals the critical role of the novel lncRNA *BIANCR* in intramuscular adipogenesis through the ERK1/2 signaling pathway

**DOI:** 10.1186/s40104-022-00820-1

**Published:** 2023-02-03

**Authors:** Xinhao Ma, Xinran Yang, Dianqi Zhang, Wenzhen Zhang, Xiaoyu Wang, Kuncheng Xie, Jie He, Chugang Mei, Linsen Zan

**Affiliations:** 1 College of Animal Science and Technology, Northwest A&F University, Yangling, Shaanxi 712100 People’s Republic of China; 2 Xi’an Dairy Cow Breeding Center, Xi’an Agriculture and Rural Bureau, Xi’an, Shaanxi 712100 People’s Republic of China; 3 National Beef Cattle Improvement Center, Northwest A&F University, Yangling, Shaanxi 712100 People’s Republic of China

**Keywords:** Adipogenesis, *BIANCR*, Bovine, Intramuscular adipocyte, LncRNAs

## Abstract

**Background:**

Long non-coding RNAs (lncRNAs) regulate numerous biological processes, including adipogenesis. Research on adipogenesis will assist in the treatment of human metabolic diseases and improve meat quality in livestock, such as the content of intramuscular fat (IMF). However, the significance of lncRNAs in intramuscular adipogenesis remains unclear. This research aimed to reveal the lncRNAs transcriptomic profiles in the process of bovine intramuscular adipogenesis and to identify the lncRNAs involved in the adipogenesis of bovine intramuscular adipocytes.

**Results:**

In this research, a landscape of lncRNAs was identified with RNA-seq in bovine intramuscular adipocytes at four adipogenesis stages (0 d, 3 d, 6 d, and 9 d after differentiation). A total of 7035 lncRNAs were detected, including 3396 novel lncRNAs. Based on the results of differential analysis, co-expression analysis, and functional prediction, we focused on the bovine intramuscular adipogenesis-associated long non-coding RNA (*BIANCR*), a novel lncRNA that may have an important regulatory function. The knockdown of *BIANCR* inhibited proliferation and promoted apoptosis of intramuscular preadipocytes. Moreover, *BIANCR* knockdown inhibited intramuscular adipogenesis by regulating the ERK1/2 signaling pathway.

**Conclusion:**

This study obtained the landscape of lncRNAs during adipogenesis in bovine intramuscular adipocytes. *BIANCR* plays a crucial role in adipogenesis through the ERK1/2 signaling pathway. The results are noteworthy for improving beef meat quality, molecular breeding, and metabolic disease research.

**Graphical Abstract:**

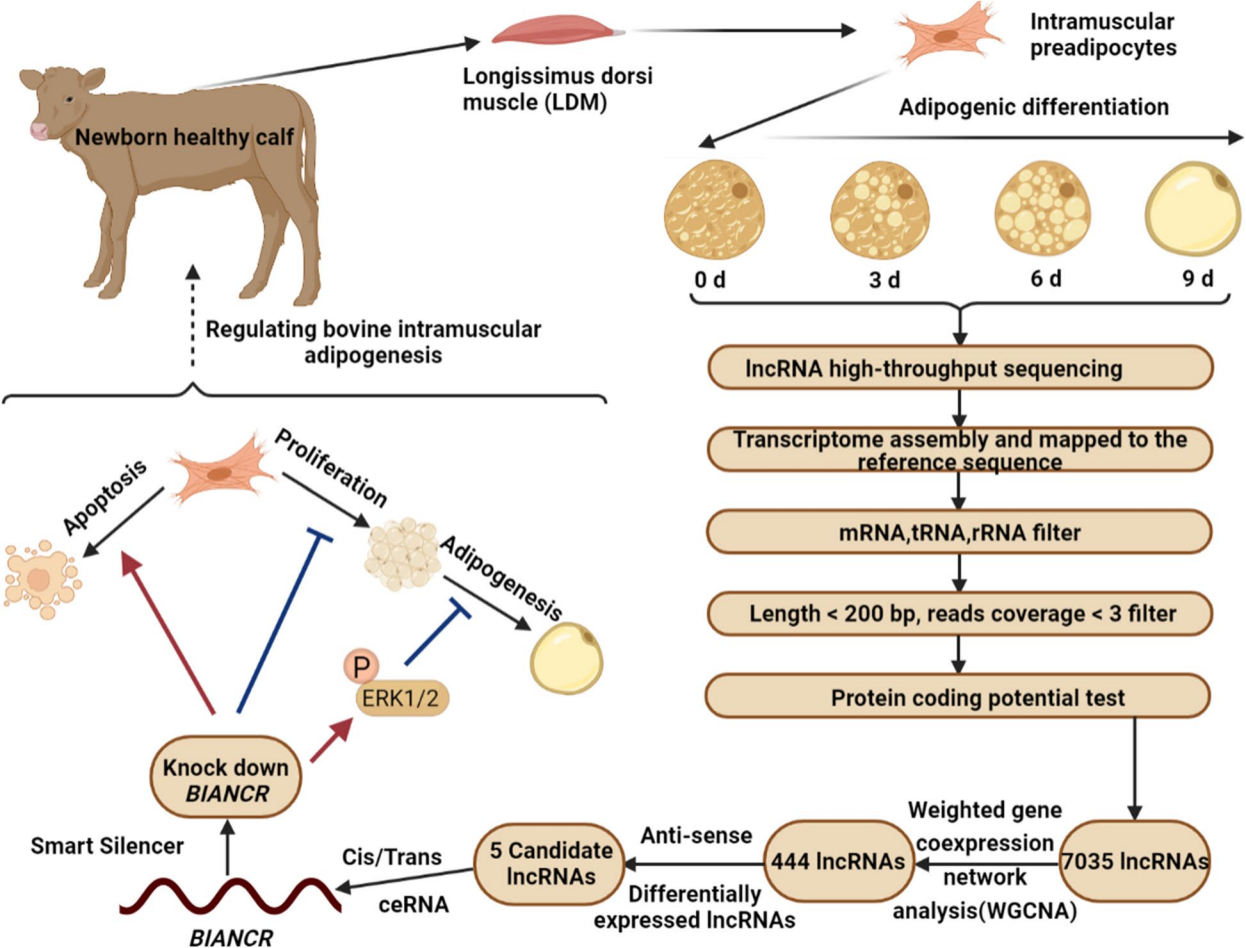

**Supplementary Information:**

The online version contains supplementary material available at 10.1186/s40104-022-00820-1.

## Introduction

People's food choices have become healthier, more flavorful, and more nutritious through rapid economic development and continuous social enhancement [1–3]. Abundant evidence reveals that intramuscular fat (IMF), as the principal component of meat marbling, can significantly improve the flavor, tenderness, and juiciness of meat products [4, 5]. And the abundance of polyunsaturated fatty acids (PUFAs) in IMF is beneficial to consumer health [6, 7]. Although IMF can considerably improve the quality of meat products, the IMF content in meat products remains low. Therefore, increasing the IMF content has become an urgent agricultural problem.

The accumulation of IMF is impacted ​by various regulatory factors in intramuscular adipocytes, such as DNA methylases, transcription factors, and noncoding RNAs [8, 9]. Long non-coding RNAs (lncRNAs) are endogenous noncoding RNAs with a length > 200 nt. Numerous studies have indicated that lncRNAs can affect IMF deposition by regulating the development of adipocytes, lipid metabolism, and fat type conversion [10–12]. A previous study utilized RNA-seq to identify 1032 lncRNAs in the longissimus dorsi muscle (LDM) of Laiwu pigs at various developmental stages. Functional annotation of lncRNAs revealed that fat cell differentiation, fatty acid degradation, and the peroxisome proliferator-activated receptor signaling pathway are involved in the generation of IMF [13]. A similar study compared the RNA-seq data of LDM from fat-type pigs and lean-type pigs, and the differentially expressed lncRNA *lnc_000414* was discovered to inhibit the proliferation of intramuscular preadipocytes [14]. However, the majority of these studies focused on monogastric animals instead of beef cattle. In particular, there is a scarcity of information on lncRNA expression patterns, functions, and mechanisms in bovine intramuscular adipogenesis.

Qinchuan beef cattle have excellent meat quality and are considered to be the best native cattle breed in China [15]. This research employed 12 biological samples of Qinchuan beef cattle intramuscular adipocytes at four adipogenesis stages (0 d, 3 d, 6 d, and 9 d after differentiation). A total of 605 differentially expressed lncRNAs (DELs) were detected utilizing RNA-seq analysis. A series of candidate lncRNAs were uncovered through bioinformatics analysis. The biological functions of the bovine intramuscular adipogenesis-associated long noncoding RNA (*BIANCR*) were identified in intramuscular adipogenesis. The objective of this research was to reveal the function and mechanism of lncRNAs in bovine intramuscular adipogenesis and establish a foundation for improving beef quality, directional selection, and beef cattle molecular breeding.

## Methods

### Ethics statement

The Committee of Experimental Animal Management at Northwest A&F University approved all animal protocols for the experiments. The model animals were utilized in compliance with the standards and rules of the organization and the government.

### Intramuscular preadipocyte isolation and sample preparation

Tissue samples were extracted from newborn (*n* = 3) and 48-month-old (*n* = 3) Qinchuan beef cattle that were fed at the National Beef Cattle Improvement Center (Xianyang, Shaanxi, China) at Northwest A&F University. The heart, liver, spleen, lung, kidney, subcutaneous fat, and *longissimus dorsi* muscle (LDM) tissues from newborn calves (*n* = 3) and 48-month-old cattle (*n* = 3) were collected with sterile surgical instruments and washed with a cold phosphate buffer (PBS). The 42 tissue samples were promptly frozen in liquid nitrogen and stored at −80 °C for RNA extraction. The tissue samples from the newborn cattle and 48-month-old cattle were collected at the same time. There are 6 instead of 3 biological replicates in tissue samples. Intramuscular preadipocyte samples were isolated from the LDM of newborn calves and inoculated into a petri dish according to previously published methods [16]. All cattle were anesthetized before slaughter.

### Culture and differentiation of intramuscular preadipocytes

The intramuscular preadipocytes were cultured in complete media (DMEM-F12 (Gibco, Grand Island, NY, USA) supplemented with 15% fetal bovine serum (PAN, Aidenbach, Germany) and 1% penicillin–streptomycin (HyClone, UT, USA)) at 37 °C and 5% CO_2_ and refreshed every 2 d. An adipogenic differentiation induction medium (complete medium containing 0.5 mmol/L 3-isobutyl-1-methylxanthine (IBMX), 1 mmol/L DXMS, and 2 mmol/L insulin) was used instead of the complete medium for induced differentiation when the intramuscular preadipocytes reached 90% confluence, and the complete medium containing 2 mmol/L insulin was shifted to maintain differentiation 2 d later.

### Oil Red O staining

The adipocytes were washed three times with PBS and fixed in 4% paraformaldehyde for 30 min after the complete medium was discarded. Next, the adipocytes were stained with Oil Red O for 30 min after being washed three times with PBS. Finally, the adipocytes were photographed by an optical microscope (Olympus, Tokyo, Japan) after being washed three times with PBS.

### RNA extraction, library preparation, and sequencing

A total of 12 intramuscular adipocyte samples (*n* = 3) were collected at four adipogenesis stages (0 d, 3 d, 6 d, and 9 d after differentiation) for the RNA-seq analysis. Total RNA was extracted and purified according to the manufacturer's instructions with TRIzol reagent (Invitrogen, Carlsbad, CA, USA). The quantity and purity of the RNA in each sample were measured by a NanoDrop ND-1000. The RNA integrity was evaluated using an Agilent 2100 (Agilent, Santa Clara, CA, USA). The samples with an RNA integrity number (RIN) > 7.0 were reserved according to the instructions of the Ribo-Zero rRNA Removal Kit (Illumina, San Diego, USA), and approximately 5 µg of total RNA was used to deplete the ribosomal RNA. Following the removal of the ribosomal RNAs, the remaining RNAs were fragmented into small fragments at high temperatures. The cleaved RNA pieces were then reverse-transcribed to generate cDNA, which was subsequently utilized to synthesize U-labeled second-stranded DNAs. The U-labeled second-stranded DNAs were treated with the heat-labile UDG enzyme after size selection by AMPureXP beads (Beckman, Brea, CA, USA). The ligated products were amplified with PCR. TruSeq Stranded Total RNA HT Sample Prep Kit (Illumina, San Diego, USA) was used to build the library. The average insert size for the final cDNA library was 300 bp (± 50 bp). Finally, we executed paired-end sequencing with an Illumina NovaSeq 6000 following the suggested technique of the manufacturer (LCBio, Hangzhou, China). Cutadapt 1.10 [17] was used to remove the reads that contained adaptor contamination, low-quality bases, and undetermined bases. Then, sequence quality was verified using FastQC 0.10.1 [18]. Twelve high-quality clean databases were obtained, the detailed information on the reads is listed in Additional file 1. The acquired reads were plotted to distinctive positions on the bovine reference genome (GCF_002263795.1_ARS-UCD1.2) with Bowtie2 [19] and Hisat2 [20]. The genome was constructed from the lung tissues of Hereford cattle (*Bos taurus*) and cited in at least 90 studies. The mapped reads of each sample were assembled with StringTie [21]. Then, the expression levels of all the transcriptomes from the 12 samples were calculate with StringTie [21] and edgeR [22].

### Identification of lncRNAs

Candidate lncRNAs were identified based on two criteria: 1. Transcripts with a length < 200 nt and reads coverage < 3 were removed to eliminate the interference of other noncoding RNAs (ribosomal RNA, transfer RNA, small nucleolar RNA, and small nuclear RNA). 2. The coding ability of the transcripts was predicted through the coding-noncoding index (CNCI) [23] and coding potential calculator (CPC) [24], and transcripts with coding potential were removed. Finally, the remaining transcripts were defined as lncRNAs. Furthermore, the novel lncRNAs were identified by sequence alignment with NONCODE [25] by BLASTN [26].

### Analysis of differentially expressed (DE) lncRNAs

The fragments per kilobase of transcript per million reads mapped (FPKM) value was calculated by StringTie [21, 27] and edgeR [22]. Then, it was used to estimate the expression levels of the lncRNAs, which with a |log_2_ (fold change)|> 1 and a false discovery rate (FDR) < 0.05 between any two adipogenesis stages (0 d vs. 3 d, 0 d vs. 6 d, 0 d vs. 9 d, 3 d vs. 6 d, 3 d vs. 9 d, and 6 d vs. 9 d after differentiation) were classified as DE lncRNAs by edgeR [22].

### Identification and functional annotation of the lncRNA target genes

The *cis*, *trans*, and indirectly regulated target mRNAs of the DE lncRNAs were annotated through Gene Ontology (GO) and Kyoto Encyclopedia of Genes and Genome (KEGG) analysis for predicting the function of the DE lncRNAs. The mRNAs that were differentially expressed and located within 100 kb upstream and downstream of the genomic location of the lncRNA were defined as *cis*-target mRNAs [28]; the mRNAs of the lncRNA-mRNA pairs with a Pearson correlation coefficient of expression levels > 0.95 and *P* < 0.05 were identified as *trans*-target mRNAs; the target mRNAs and lncRNAs of the miRNAs were predicted using the miRanda [29] program (miRanda Energy < −20 kcal/mol) and TargetScan [30] online tools (TargetScan score ≥ 90). The mRNAs and lncRNAs targeted by the same miRNA were considered to be involved in a ceRNA network. The GO and KEGG analyses of the above mRNAs used the DAVID [31] 6.8 bioinformatics resources and the KOBAS 3.0 database [32], respectively. Terms or pathways with a *P* < 0.05 and more than 2 matching genes were identified as significant. Finally, the regulatory network of lncRNAs with their target genes was constructed using the Cytoscape software [33].

### Weighted gene co-expression network analysis (WGCNA)

The lncRNAs were separated into distinct modules using cluster analysis of their expression levels, and the relationships between each module and adipogenesis stages were established by phenotypic correlation. To identify critical lncRNAs in the co-expression network, the correlation coefficients between each lncRNA and its module and adipogenesis stages were evaluated. The input is a lncRNA expression matrix, and the process was accomplished using the R script WGCNA [34].

### Nuclear and cytoplasmic RNA separation

Nuclear and cytoplasmic RNA of the intramuscular adipocytes were separated with a total protein and RNA separation kit (Invitrogen, Carlsbad, CA, USA). The nuclear and cytoplasmic RNAs were reverse transcribed after being quantified by 1% agarose gel electrophoresis. Finally, quantitative real-time PCR (qRT-PCR) was performed to determine the subcellular localization of the lncRNAs. The primer information is listed in Additional file 2.

### Transient transfection of intramuscular adipocytes

According to the manufacturer's instructions, the Smart Silencer *BIANCR* and Smart Silencer negative control (RiboBio, Guangzhou, China) were transfected into the intramuscular preadipocytes using Liposome 3000 (Invitrogen, Carlsbad, CA, USA). Then, the medium was changed to a complete medium after 8 h, and the interference efficiency was evaluated with qRT-PCR after 2 d.

### qRT–PCR analysis

Total RNA was extracted from the tissues and adipocytes according to the protocol of the RNAiso Plus Kit (Takara, Beijing, China). The qualities and quantities of total RNA were evaluated with an ultramicro spectrophotometer (TECAN, Zurich, SUI) and 1% agarose gel electrophoresis. The RNA samples were reverse transcribed with the Prime Script RT Reagent Kit (Takara, Beijing, China). The qRT-PCR analysis was completed with TB Green Premix Ex Taq II (Takara, Beijing, China) and the CFX Connect Real-Time PCR Detection System (Bio-Rad, Hercules, California, USA). The expression levels of mRNAs and lncRNAs were quantified using the 2^−ΔΔCT^ method. Actin beta (*ACTB*) was used as the internal control, and primer information is listed in Additional file 2.

### Western blotting

Total protein was extracted from the intramuscular adipocytes with a WB/IP lysis buffer supplemented with 1% PMSF (Beyotime, Shanghai, China). According to molecular weight, 15 μg of total protein was separated by polyacrylamide gel electrophoresis. The proteins on the PVDF membrane were blocked for 30 min with the Quick Block Western Blocking Solution (Beyotime, Shanghai, China). Subsequently, the PVDF membranes were incubated with primary antibodies and secondary antibodies for 12 h and 2 h at 4 ℃ and RT, respectively. Finally, a DocTMXR system (Bio-Rad, Hercules, California, USA) was utilized to expose the protein bands. The protein on the PVDF membranes was flushed with a 1 × TBST buffer in the above steps. Antibody information is provided in Additional file 3.

### EdU staining and CCK-8 cell proliferation

EdU staining and CCK-8 cell proliferation assays were performed on the intramuscular preadipocytes with a YF 594 Click-iT EdU Kit (UElandy, Suzhou, China) and a Cell Counting Kit (UElandy, Suzhou, China) 48 h after transfection, respectively [35]. The relevant processes were performed according to the manufacturer’s protocols.

### Triacylglycerol (TAG) assay

The extraction of TAG from intramuscular adipocytes was performed with a Cell/Tissue Triglyceride Determination Kit (Applygen, Beijing, China). The TAG content was measured at 550 nm [36]. The relevant processes were performed in accordance with the manufacturer’s protocols.

### Flow cytometry (FCM)

The intramuscular preadipocytes were treated according to the protocol of the Cell Cycle Staining Kit (Multisciences, Hangzhou, China) and the Annexin V-FITC/PI Apoptosis Kit (Multisciences, Hangzhou, China) 48 h after transfection. A flow cytometer (CytoFLEX, Beckman, Brea, CA, USA) was used to count 10,000 cells to investigate the change in the cell cycle and apoptosis.

### Statistical analysis

The data are presented as the mean ± standard deviation (SD). GraphPad Prism 9.0.2 (GraphPad Software, San Diego, California, USA) was utilized to perform statistical analysis and construct images. *P* values were calculated using one-way analysis of variance (ANOVA), Dunnett's multiple comparison test and Student’s *t*-test (in two group data). The significance levels ^**^P < 0.01 or ^*^P < 0.05 defined the differences as either very significant or significant.

## Results

### RNA-Seq analysis of the intramuscular adipocytes during adipogenesis

**Fig. 1:**
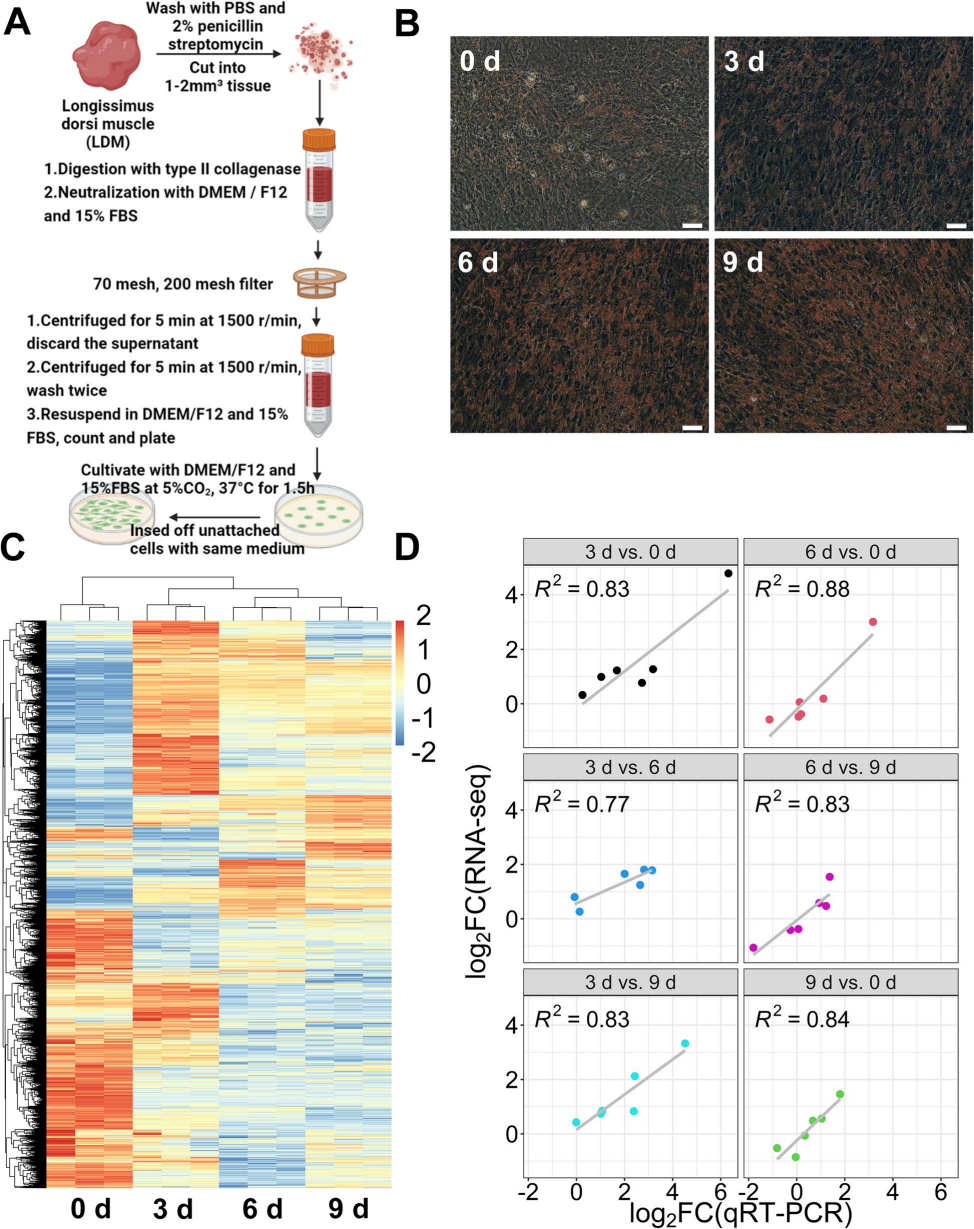
RNA-Seq of intramuscular adipocytes during adipogenesis **A** Intramuscular adipocytes were isolated from the LDM. **B** Oil Red O staining of intramuscular adipocytes at four adipogenesis stages (scale bar: 50 μm). **C** Heatmap presented all lncRNAs in intramuscular adipocytes during adipogenesis. **D** The correlation of RNA-seq (*y*-axis) with qRT-PCR data (*x*-axis) using the log_2_(fold change) measure of the adipogenesis related genes in pairwise comparison of four adipogenesis stages

To reveal the role of lncRNAs in the intramuscular adipogenesis, high-throughput sequencing technology was applied to the intramuscular adipocytes at four adipogenesis stages (0 d, 3 d, 6 d, and 9 d after differentiation). The isolation of the intramuscular preadipocytes was performed based on previous experiments (Fig. 1A). The number of lipid droplets stained with Oil Red O increased as the differentiation process continued (Fig. 1B), which confirmed the growth status and differentiation capacity of intramuscular adipogenesis. A total of 7035 lncRNAs were acquired across four adipogenesis stages (Fig. 1C). The mRNA expression levels of peroxisome proliferator-activated receptor gamma (*PPARG*), CCAAT enhancer binding protein alpha (*CEBPA*), CCAAT enhancer binding protein beta (*CEBPB*), sterol regulatory element binding transcription factor 1 (*SREBF1*), fatty acid binding protein 4 (*FABP4*), and lipoprotein lipase (*LPL*) were detected to evaluate the data reliability of the RNA-seq. The results of the qRT-PCR analysis showed a similar expression trend as the RNA-seq analysis, which demonstrated the strong consistency between qRT-PCR and RNA-Seq techniques (Fig. 1D, Additional file 4). The above data indicate that the differentiation of intramuscular adipocytes and the RNA-seq process are both normal and can be explored further.

### Characteristics of lncRNAs during intramuscular adipogenesis

**Fig. 2:**
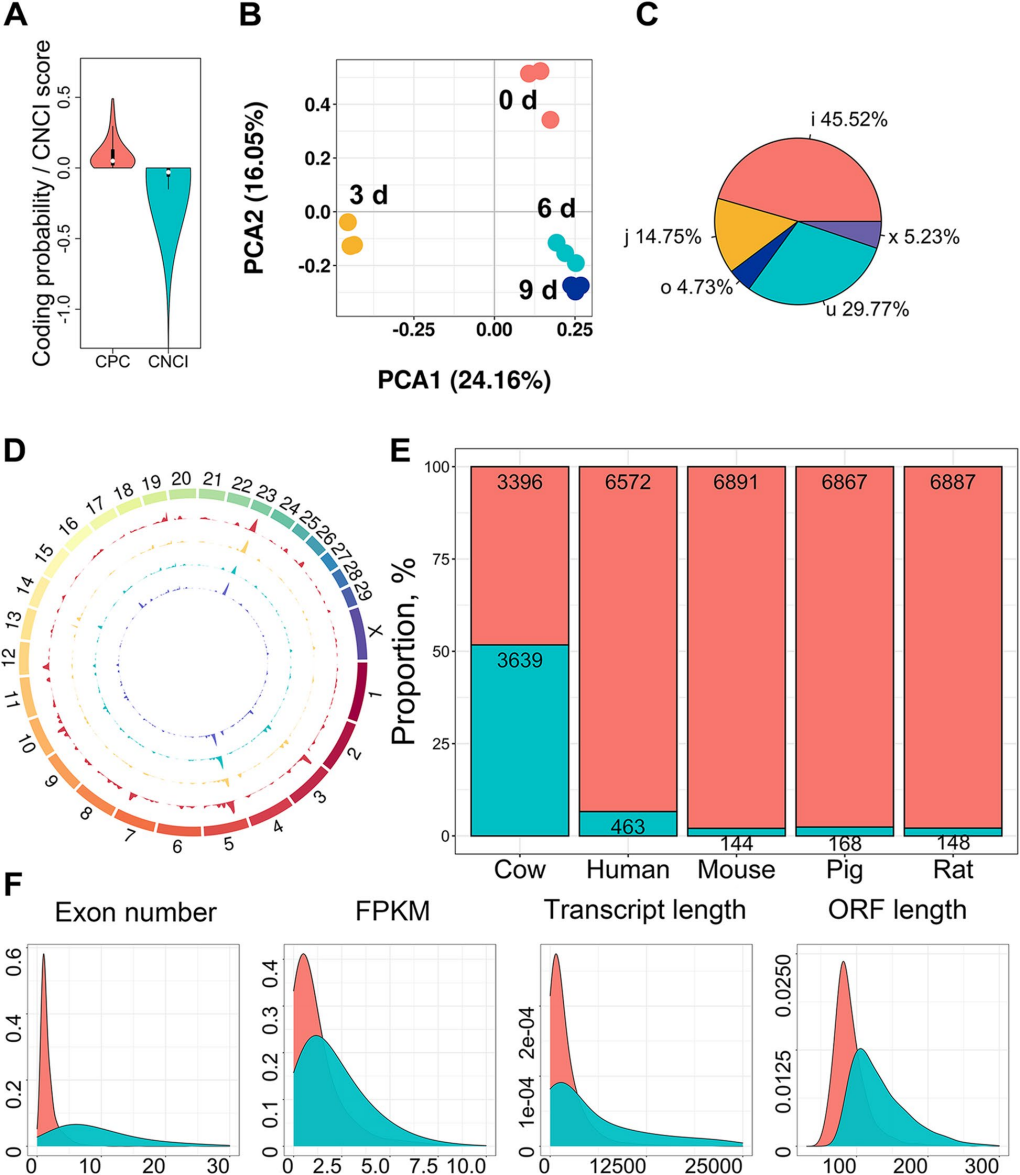
Characters of lncRNAs during intramuscular adipogenesis **A** Coding potential of candidate lncRNAs was evaluated with CPC, and CNCI. **B** Principal component analysis (PCA) for lncRNAs in 12 samples. **C** The type and percentage of lncRNAs (the meaning of the letters was listed in Additional file 6). **D** The Circos diagram showed that lncRNAs distributed in different chromosomes at various adipogenesis stages. The circles from outside to inside represent 0 d, 3 d, 6 d, and 9 d respectively. **E** In all lncRNAs, the quantity and proportion of novel lncRNAs at cattle and conserved lncRNAs at pigs, humans, rats and mouse. The pink and cyan represents novel/un conserved and known/conserved lncRNA respectively. **F** Exon number, expression levels, transcript length and open reading frame (ORF) length distribution of all lncRNAs and mRNAs. The pink and cyan represents lncRNA and mRNA respectively

Transcripts with lengths > 200 nt and reads coverage ≥ 3 were evaluated for their protein-coding ability. Transcripts with a coding probability < 0.5 and CNCI scores < 0 were classified as lncRNAs (Fig. 2A). The sample correlation analysis and principal component analysis (PCA) demonstrated that the expression of the lncRNAs varied across different adipogenesis stages (Fig. 2B, Additional file 5), and the results of the RNA-seq analysis could be employed for differential analysis. The lincRNAs accounted for the highest number of lncRNAs (Fig. 2C, Additional file 6). Compared to other species, the distribution of the identified lncRNAs across chromosomes was not homogeneous (Fig. 2D), and the sequence-conserved lncRNAs accounted for the largest proportion in the cattle tissues (Fig. 2E). The above results are consistent with the existing understanding of lncRNAs. Furthermore, the transcript features of the lncRNAs were also consistent with the existing scientific understanding. The transcript lengths of the lncRNAs were shorter than that of mRNAs, and the number of exons, ORF length, and expression levels were similar (Fig. 2F). These discoveries expand our knowledge of lncRNAs in bovine intramuscular adipocytes.

### DELs during intramuscular adipogenesis

**Fig. 3:**
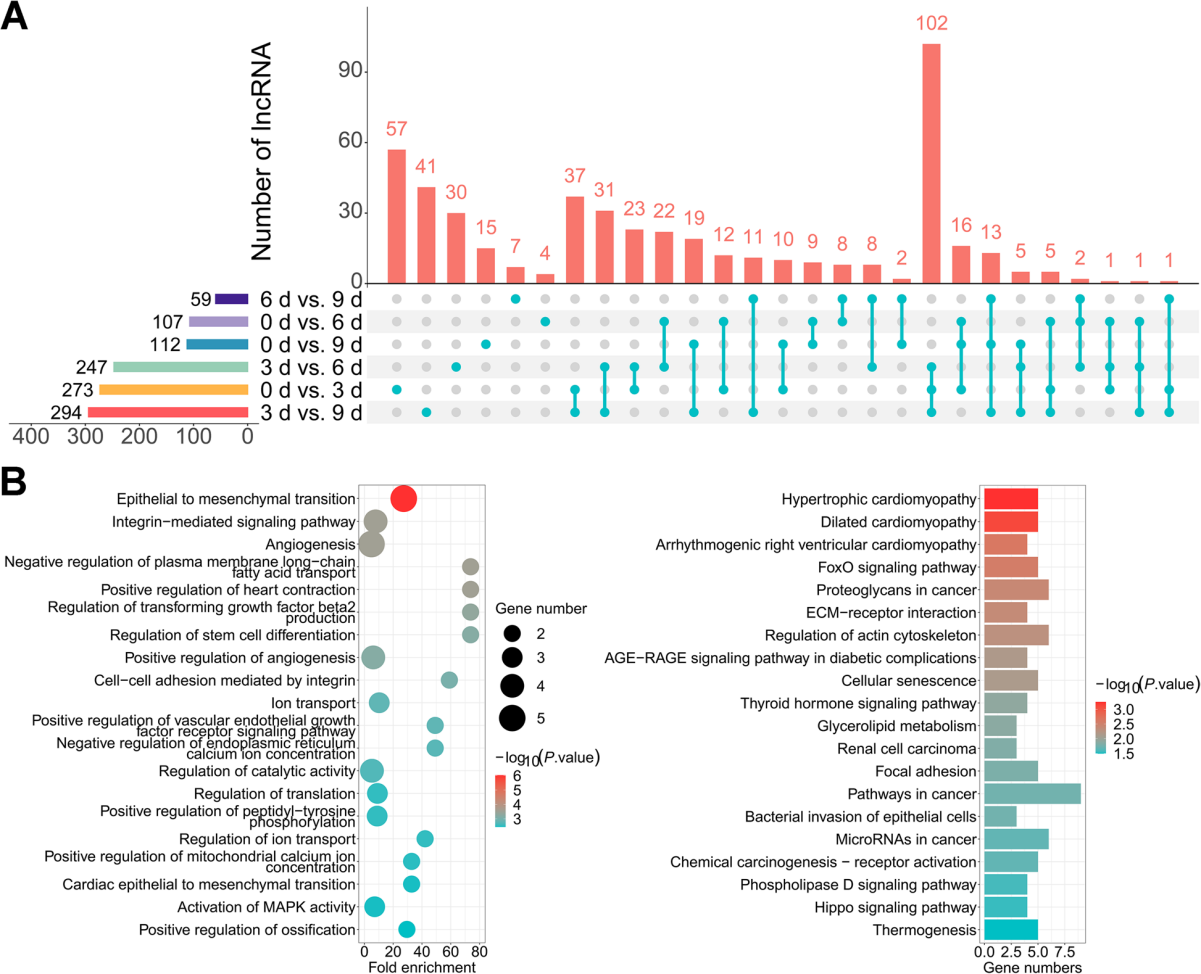
DELs during intramuscular adipogenesis **A** The number of DELs at four adipogenesis stages. **B** GO (left) and KEGG (right) analysis of DELs. The top 20 enriched terms or pathways are showed according to *P*-values

The functions of the lncRNAs in the intramuscular adipogenesis were investigated. There were 273, 107, 112, 247, 294, and 59 lncRNAs identified as DELs among the 6 comparison groups (Fig. 3A, Additional file 7). The GO terms and KEGG signaling pathways of the target genes of these DELs were related to the negative regulation of plasma membrane long-chain fatty acid transport, the regulation of stem cell differentiation, the activation of MAPK activity, the FoxO signaling pathway, and glycerolipid metabolism (Fig. 3B, Additional file 8).

### Co-expression network analysis of lncRNAs

**Fig. 4:**
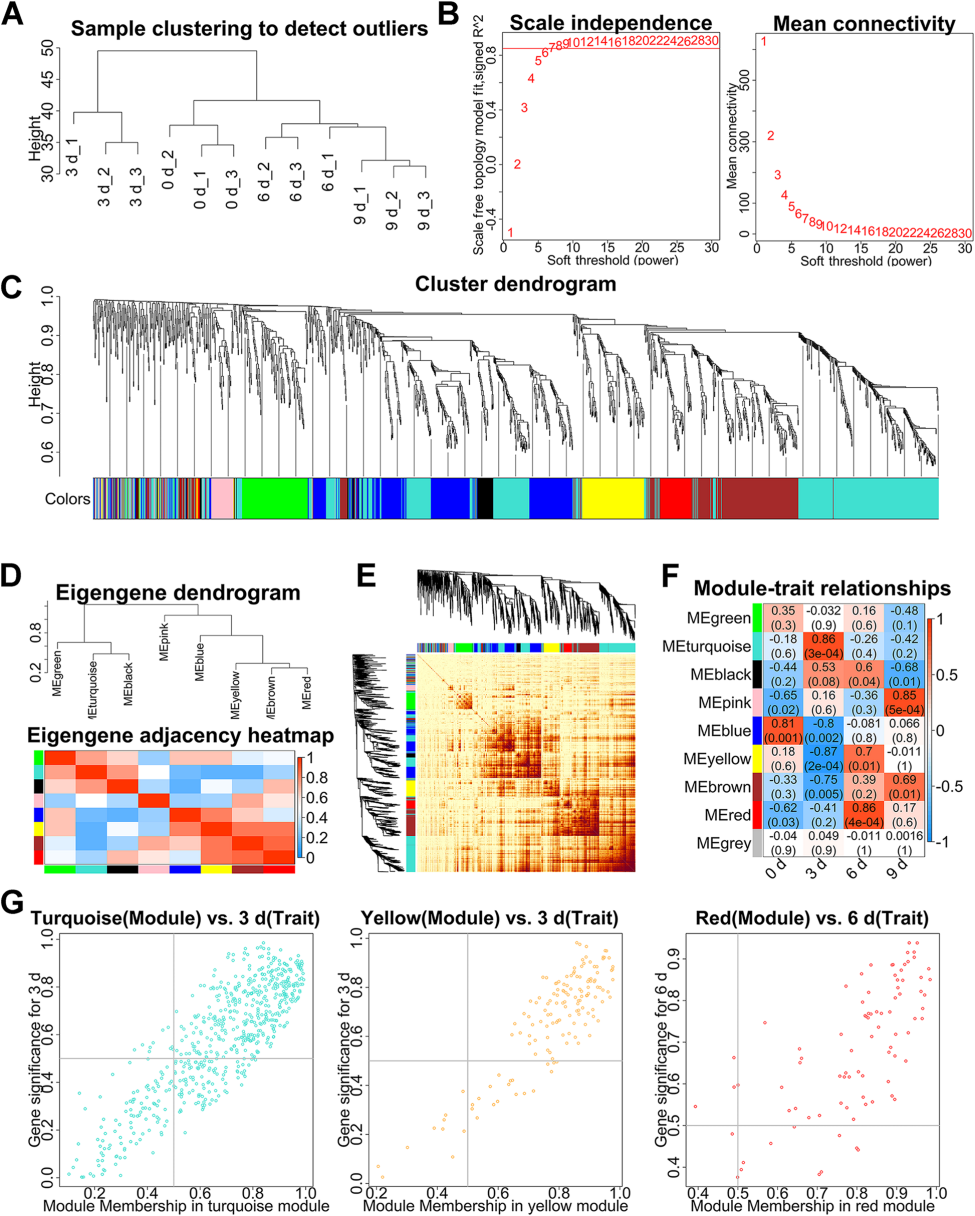
Co-expression network analysis of lncRNAs **A** Hierarchical clustering tree of all samples. **B** The determination of soft thresholding power (β = 7) by scale free topology model fit (left) and mean connectivity (right). **C** The clustering dendrogram demonstrates lncRNAs were separated into various modules based on the dynamic hybrid-cutting method. **D** The eigengene adjacency heatmap with the module clustering tree (above) and the corresponding module clustering heatmap (below). **E** Topological overlap matrix for all lncRNAs in each module. **F** Module-trait relationship heatmap exhibiting the correlation coefficient between each module and the four adipogenesis stages. **G** The Gene Module Membership and the gene Trait Cor scattering distribution of turquoise vs. 3 d (left), yellow vs. 3 d (middle), and red vs. 3 d (right)

All samples were subjected to co-expression analysis after removing outliers by a hierarchical clustering tree (Fig. 4A). The correlation coefficient with a scale-free topology model fit > 0.85 and mean connectivity < 100 was determined as the soft threshold (β = 7) (Fig. 4B). Here, the lncRNAs with at least 75% similarity in their expression pattern were clustered into modules with the same color according to the soft threshold (Fig. 4C). The results of the correlation analysis between modules and the lncRNAs revealed that each module was comparatively separate from the others, and the lncRNAs in the same module were strongly connected, indicating that the clustering of the lncRNAs was effective (Fig. 4D–E). We constructed correlation heatmaps for each module and different adipogenesis stages (Fig. 4F). Three module-adipogenesis stage pairs (turquoise-3 d, yellow-3 d, and red-3 d) with correlation coefficients > 0.8 were labeled essential modules. The lncRNAs in four modules with both gene Module Membership and gene TraitCor > 0.5 were identified as critical lncRNAs in the corresponding stage of differentiation (Fig. 4G, Additional file 9).

### Identification of *BIANCR* as a candidate lncRNA

**Fig. 5:**
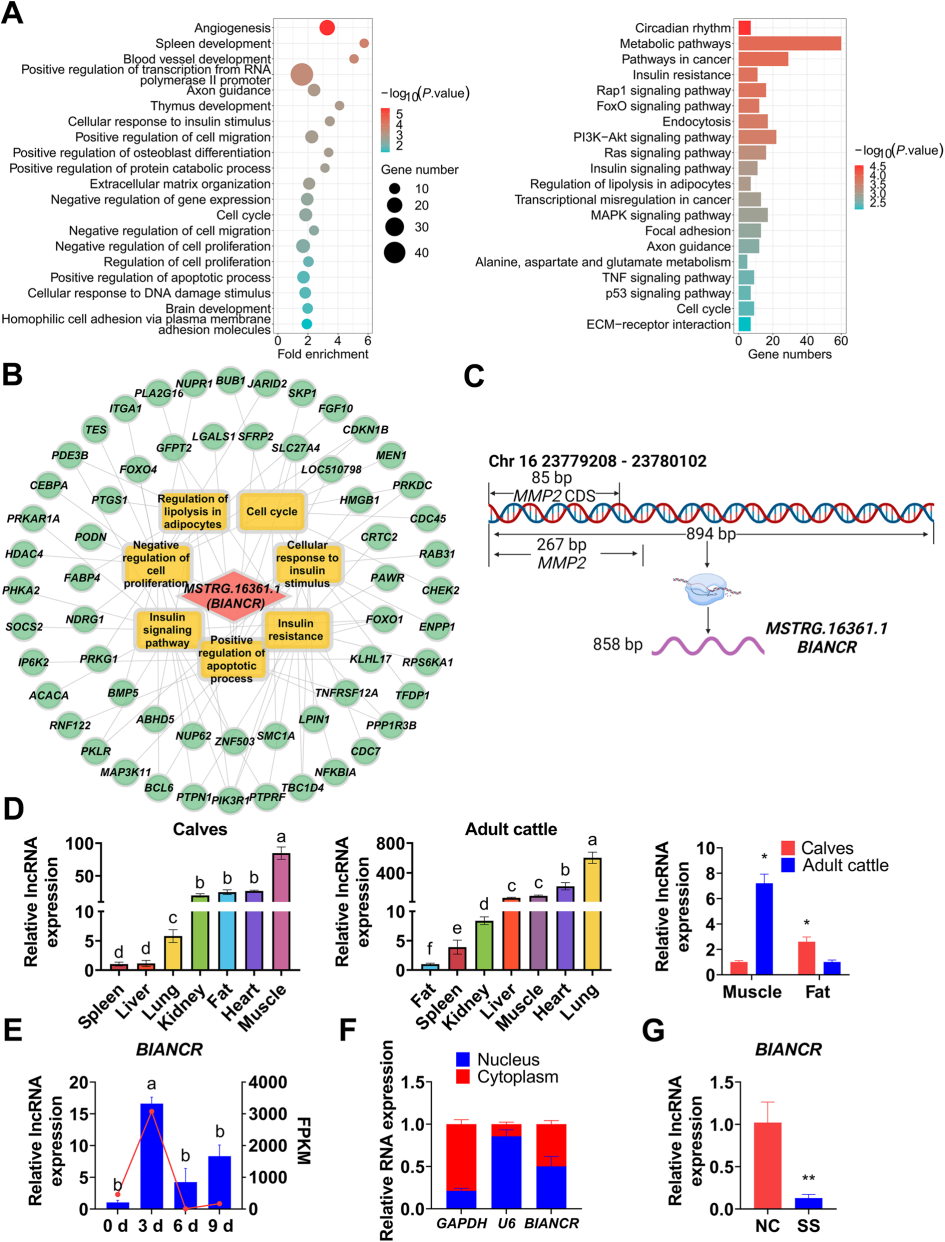
Identification of *BIANCR* as a candidate lncRNA **A** GO (left) and KEGG (right) analysis of lncRNA *MSTRG.16361.1*. The top 20 enriched terms or pathways are showed according to *P*-values. **B** The potential regulatory network of lncRNA *MSTRG.16361.1*. Pink diamond, yellow rectangle, and cyan circle represents lncRNA, GO term/KEGG pathway, and Genes respectively. **C** Chromosome location and transcriptional structure of *BIANCR*. **D** The expression levels of *BIANCR* in various tissues of calves (left), adult cattle (mid) and comparison (right) between calves and adult cattle. **E** The expression levels of *BIANCR* in different adipogenesis stage. **F** qRT-PCR detection of *BIANCR* in the cytoplasmic and nuclear fractions of Intramuscular adipocytes. *U6* and glyceraldehyde-3-phosphate dehydrogenase (*GAPDH*) provide as cytoplasmic and nuclear localization control respectively. **G** The knockdown of *BIANCR* utilizing RNA interference. Results are presented as the means ± SD, *n* = 3, *P < 0.05; ** *P* < 0.01; different lowercase letters indicate significant differences (*P* < 0.05)

Differentially expressed and antisense critical lncRNAs were identified for subsequent research. Interestingly, the GO terms and KEGG signaling pathways of the target genes of lncRNA *MSTRG.16361.1* were related to the cell cycle, the regulation of cell proliferation, the positive regulation of the apoptotic process, insulin resistance, the insulin signaling pathway, the regulation of lipolysis in adipocytes, and the FoxO signaling pathway (Fig. 5A–B, Additional files 10–11). *MSTRG.16361.1* was located on bovine chromosome 16 and had a 267 bp overlap with the 5' end of matrix metallopeptidase 2 (*MMP2*) on the sense strand (Fig. 5C, Additional file 12). The tissue expression results showed that the lncRNA *MSTRG.16361.1* was highly expressed in LDM, heart, and fat tissues from the calves and the lung, heart, and LDM tissues from adult cattle (Fig. 5D). Furthermore, the expression of lncRNA *MSTRG.16361.1* was significantly upregulated during the initial adipogenesis stages (Fig. 5E). These results suggested that the lncRNA *MSTRG.16361.1* is crucial to adipogenesis of bovine intramuscular adipocytes. The lncRNA *MSTRG.16361.1* is located in the nucleus and cytoplasm (Fig. 5F). Smart Silencer (a mixture of small interfering RNA and antisense oligonucleotide for simultaneous interfering with lncRNAs in the nucleus and cytoplasm) was utilized to interfere with *MSTRG.16361.1*, and the expression level of *MSTRG.16361.1* was decreased by 87% after 48 h (Fig. 5G). Therefore, we concentrated on *MSTRG.16361.1* and named it bovine intramuscular adipogenesis associated long non-coding RNA (*BIANCR*) in subsequent research [37].

### *BIANCR* promotes the proliferation of intramuscular preadipocytes

**Fig. 6:**
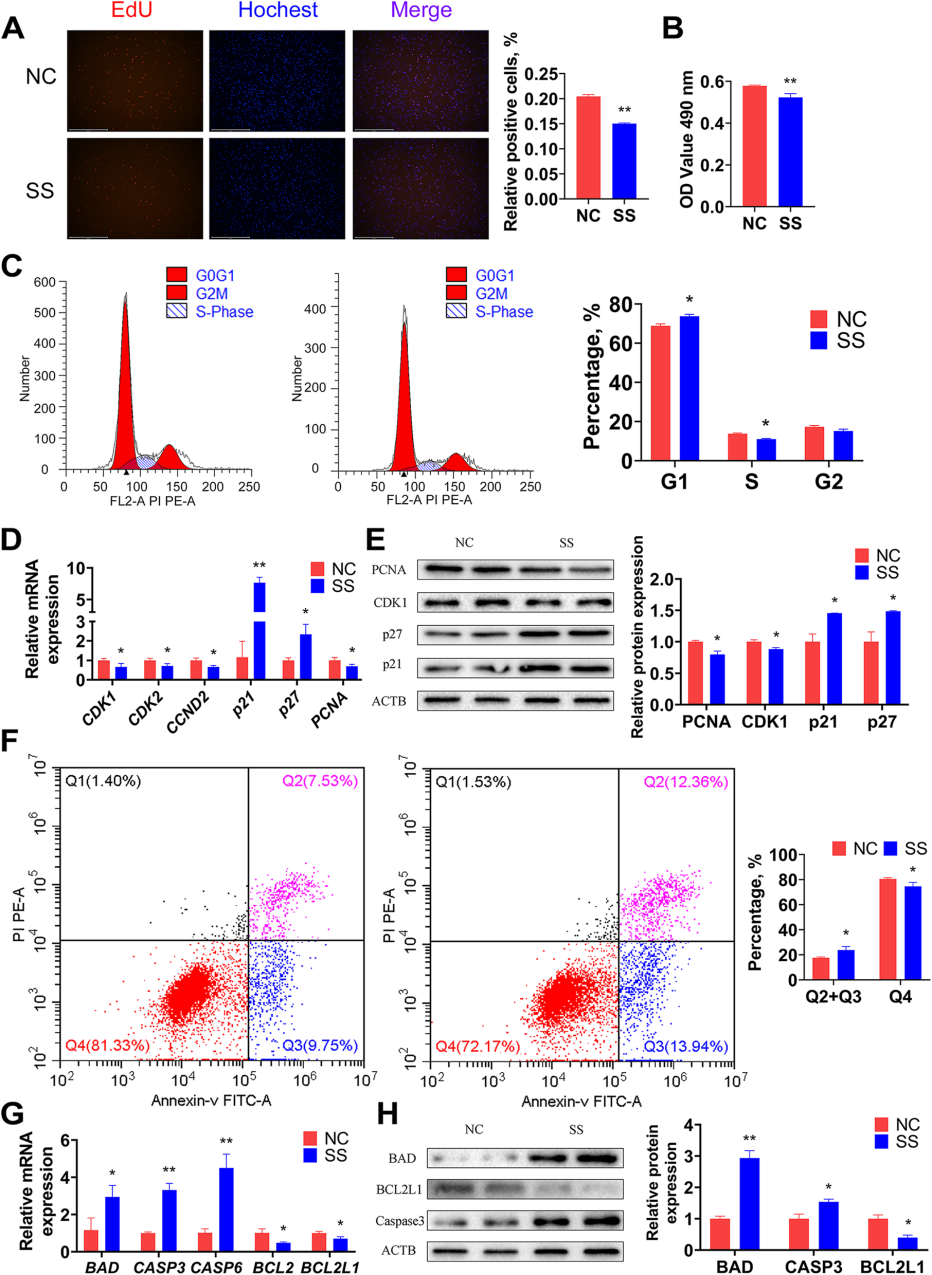
*BIANCR* promoted the proliferation and inhibited the apoptosis of intramuscular preadipocytes **A** EdU staining measured the number of intramuscular preadipocytes at proliferative stages. **B** Cell vitality was evaluated by CCK-8 assay. **C** The number of cells in G1(Gap 1 phase), S (Synthesis phase), and G2 (Gap 2 phase) were calculated by flow cytometry. **D** The mRNA expression levels of cell cycle related genes (*CDK1*, *CDK2*, *CCND2*, *PCNA*, *p21*, and *p27*). **E** The protein expression levels of cell cycle related genes (CDK1, PCNA, p21, and p27). **F** The number of viable non-apoptotic cells, viable apoptotic cells, non-viable apoptotic cells, and non-viable cells were counted by flow cytometry. **G** The mRNA expression levels of apoptotic related genes (*BAD*, *CASP3*, *CASP6*, *BCL2* and *BCL2L1*). (**H**) The protein expression levels of apoptotic related genes (BAD, CASP3, and BCL2L1). Results are presented as the means ± SD, *P < 0.05; ** *P* < 0.01

The intramuscular preadipocytes at 50% confluence were transfected with a Smart Silencer *BIANCR* (SS) and a Smart Silencer negative control (NC) to confirm the function of *BIANCR* in proliferation. At 48 h after transfection, EdU staining showed that the knockdown of *BIANCR* reduced the proportion of EdU-positive cells (Fig. 6A). The CCK8 assay indicated that the knockdown of *BIANCR* significantly inhibited intramuscular preadipocyte viability (Fig. 6B), and the FCM assay revealed that the knockdown of *BIANCR* hampered the transition from the G1 to the S phase (Fig. 6C). Furthermore, the knocking down *BIANCR* significantly decreased the mRNA expression levels of cyclin-dependent kinase 1 *(CDK1*), cyclin-dependent kinase 2 (*CDK2*), cyclin D2 (*CCND2*) and proliferating cell nuclear antigen (*PCNA*). The knockdown also significantly increased the mRNA expression levels of cyclin-dependent kinase inhibitor 1A (*p21*) and cyclin-dependent kinase inhibitor 1B (*p27*) (Fig. 6D), which was consistent with the variation in protein expression levels (Fig. 6E). These results indicated that *BIANCR* promoted the proliferation of intramuscular preadipocytes.

### *BIANCR* inhibits the apoptosis of intramuscular preadipocytes

The influence of *BIANCR* on the apoptosis of intramuscular preadipocytes was investigated further. The FCM assay revealed that the knockdown of *BIANCR* increased the proportion of apoptotic cells (Fig. 6F). Knocking down *BIANCR* significantly increased the mRNA expression levels of BCL2-associated agonist of cell death (*BAD*), cysteinyl aspartate specific proteinase-3 (*CASP3*), and cysteinyl aspartate specific proteinase-6 (*CASP6*) (Fig. 6G), while it significantly decreased the mRNA expression levels of BCL2 apoptosis regulator (*BCL2*) and BCL2 like 1 (*BCL2L1*), which was consistent with the variation in protein expression levels (Fig. 6H). These results indicated that *BIANCR* inhibited the apoptosis of intramuscular preadipocytes.

### *BIANCR* promotes the differentiation of intramuscular adipocytes through the ERK1/2 signaling pathway

**Fig. 7:**
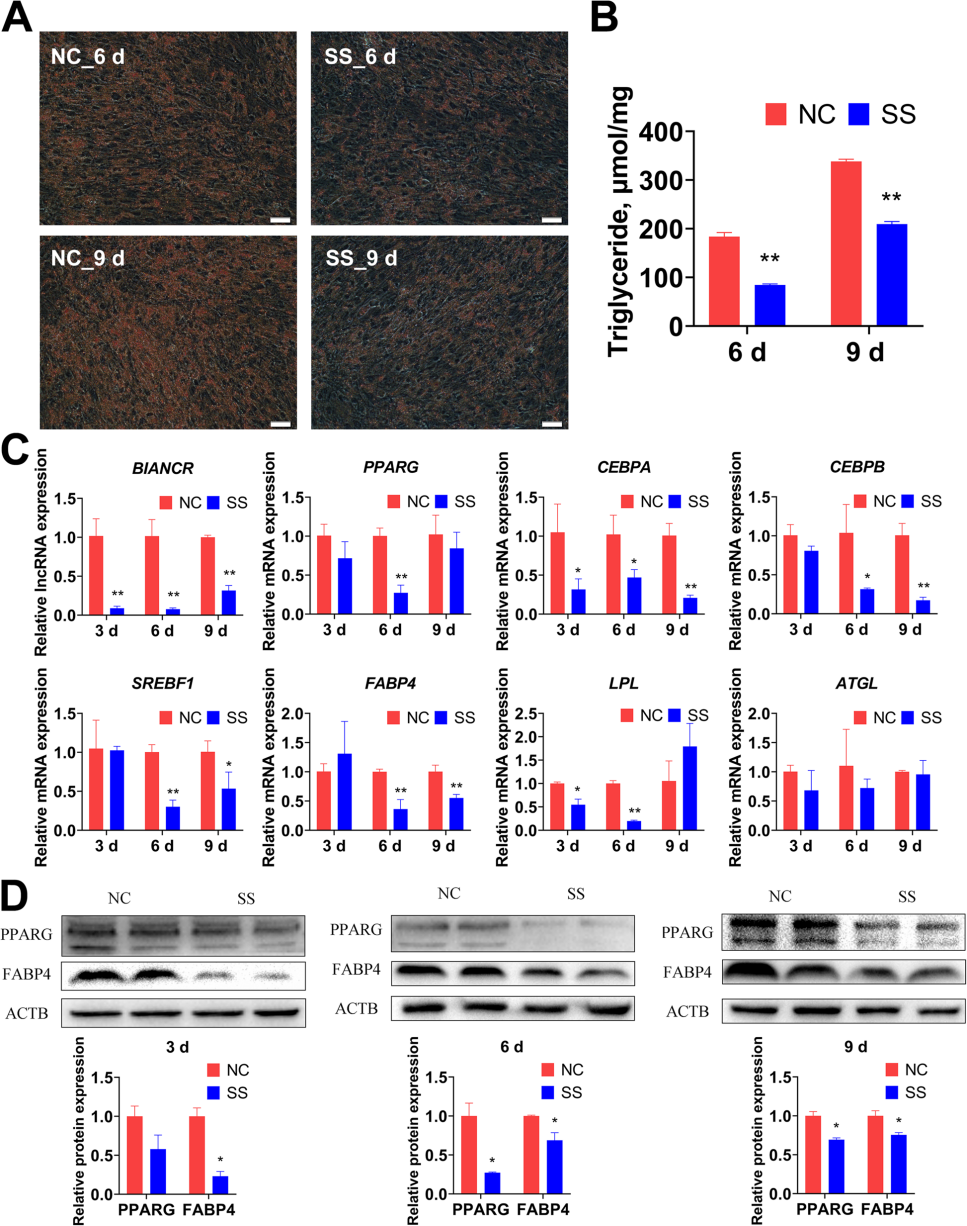
*BIANCR* promoted the differentiation of intramuscular adipocytes **A** Oil red O staining evaluated the lipid droplet content of intramuscular adipocytes (scale bar: 50 μm). **B** Triacylglycerol content of intramuscular adipocytes were measured by triacylglycerol assay. **C** The mRNA expression levels of *BIANCR* and adipogenesis related genes (*PPARG*, *CEBPA*, *CEBPB*, *SREBF1*, *FABP4*, *LPL*, and *ATGL*). **D** The protein expression levels of adipogenesis related genes (PPARG and FABP4). Results are presented as the means ± SD, *P < 0.05; ** *P* < 0.01

The intramuscular adipocytes at 90% confluence were transfected with SS and NC to clarify the effects of *BIANCR* during adipogenesis. Oil Red O staining revealed that the knockdown of *BIANCR* inhibited lipid accumulation at 6 d and 9 d after differentiation (Fig. 7A). The triacylglycerol content had consistent results (Fig. 7B). The knocking down of *BIANCR* significantly decreased the expression levels of *BIANCR* at three time points and significantly decreased the expression levels of *PPARG*, *CEBPA*, *CEBPB*, *SREBF1*, *FABP4* and *LPL* on at least one time points. However, adipose triglyceride lipase (*ATGL*) showed no noticeable changes at the mRNA level (Fig. 7C). The protein expression levels of PPARG and FABP4 were similar to the mRNA expression levels (Fig. 7D). These observations demonstrated that *BIANCR* promoted the adipogenesis of intramuscular adipocytes.

**Fig. 8:**
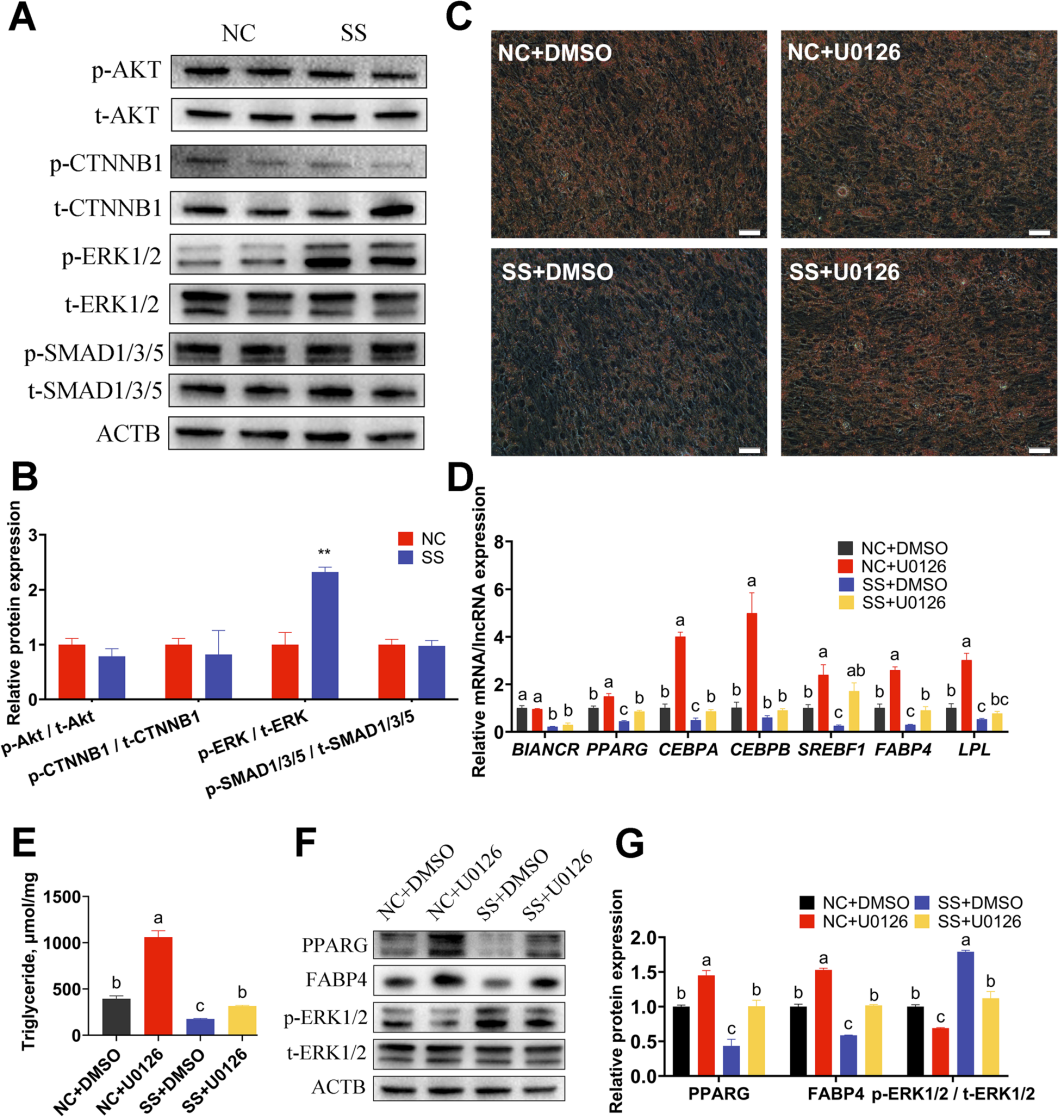
The ERK inhibitor U0126 reverses adipogenesis inhibition induced by *BIANCR* knockdown **A** The protein expression levels of AKT, p-AKT, CTNNB1, p-CTNNB1, ERK1/2, p-ERK1/2, SMAD1/3/5, and p-SMAD1/3/5. **B** Quantitative of protein expression levels. **C** Oil Red O staining determined the amount of lipid droplets in intramuscular adipocytes (scale bar: 50 μm). **D** The mRNA expression levels of *BIANCR* and adipogenesis-related genes (*PPARG*, *CEBPA*, *CEBPB*, *SREBF1*, *FABP4*, and *LPL*). **E** The content of triacylglycerol in intramuscular adipocytes were evaluated utilizing triacylglycerol assay. **F** The protein expression levels of adipogenesis-related genes (PPARG and FABP4), ERK1/2 and p-ERK1/2. **G** Quantitative of protein expression levels. Results are presented as the means ± SD, *P < 0.05; ** *P* < 0.01; different lowercase letters indicate significant differences (*P* < 0.05)

To investigate the regulatory mechanism of *BIANCR* in adipogenesis of the intramuscular adipocytes, we detected the activation of crucial proteins in the Wnt/beta-catenin, PI3K-Akt, TGF-beta/SMAD, and ERK1/2 signaling pathways according to related research on adipogenesis and KEGG analysis of *BIANCR*. The results showed that knocking down *BIANCR* significantly increased the phosphorylation level of ERK1/2 (Fig. 8A–B). Subsequently, to confirm that *BIANCR* affects intramuscular adipogenesis through the ERK1/2 signaling pathway, we knocked down *BIANCR* in the intramuscular adipocytes and treated them with U0126 (a selective ERK1/2 inhibitor, 10 μmol/L). The ratio of p-ERK/t-ERK was significantly lowered at 12 h after treatment with U0126 (Fig. 8F–G). The U0126 treatment prevented the negative effect of lipid accumulation caused by the *BIANCR* knockdown (Fig. 8C). The triacylglycerol content had consistent results (Fig. 8E). Furthermore, the qRT-PCR analysis demonstrated that U0126 prevented the downregulation of adipogenic mRNA expression levels resulting from the *BIANCR* knockdown (Fig. 8D), which was similar to the variation in protein expression levels (Fig. 8F–G). These results suggest that *BIANCR* regulated adipogenesis through the ERK1/2 pathway.

## Discussion

With the persistent evolution and widespread application of RNA-seq technologies, there are numerous recent studies on protein-coding genes, which account for only 2% of the mammalian genome [38]. Recent studies have revealed that ncRNAs are also worth investigating [39], and lncRNAs have been reported to regulate various biological processes at the epigenetic, transcriptional, and translational levels [40–42]. These discoveries have sparked interest in lncRNA research in medical hygiene, drug discovery, and agricultural production. IMF has played a vital role in the improvement of meat quality [43, 44]. A total of 11,247 lncRNAs were discovered using RNA-seq in chicken subcutaneous fat and IMF, and the regulatory functions of the novel lncRNA *lncAD* were related to IMF development [45]. In addition, bioinformatics analysis revealed that three codifferentially expressed lncRNAs in yak muscle and adipose tissues potentially regulate IMF deposition through the ceRNA network [46]. Although numerous studies have reported 23,515 lncRNAs in beef cattle [25], publications on intramuscular adipogenesis and native cow breeds are still sparse. In view of the low conservation of lncRNAs within species and tissues, it is difficult to connect similar research with IMF deposition in beef cattle. As a result, it is essential to conduct targeted research on certain developmental stages of specific tissues in individual species.

In this research, intramuscular preadipocytes isolated from the LDM of Qinchuan beef cattle were induced for adipogenesis. The landscape of the lncRNA was determined during four adipogenesis stages (0 d, 3 d, 6 d and 9 d after differentiation). Generally, the identification of lncRNAs includes transcript length and coding potential. The difference of this study is that it filtered transcripts according to reads coverage. Reads coverage reflects the breadth of each position of the transcript covered by acquired reads. This filter reduces the false-positive probability of transcripts. After removing transcripts with reads coverage < 2 or length < 200 nt, coding ability estimates identified 7035 lncRNAs, including 3396 novel lncRNAs [25]. The expression and structural characteristics of the lncRNAs in the intramuscular adipocytes were revealed through a comparative analysis between mRNAs and lncRNAs. The results were consistent with other studies [11, 14, 45]. The changes in the mRNA and lncRNA expression levels showed that 3 d is a crucial phase in determining adipogenesis, as a large number of transcripts were drastically activated or inhibited during this time point. This time point is significantly later than that of other adipocytes [47], just as intramuscular adipose tissues develop later than other adipose tissues [48]. This may be an important feature of IMF development. However, major phenotypic alterations occurred at 6 d rather than 3 d, which could be attributable to the time consumption in the translation and function of proteins; it is one of the reasons for time point selection in subsequent experiments simultaneously. This work contributes to the knowledge of IMF development and provides numerous lncRNA resources.

To identify the critical lncRNAs during adipogenesis, a total of 605 DELs were detected at four adipogenesis stages. Enrichment analysis revealed that the DELs were involved in the negative regulation of plasma membrane long-chain fatty acid transport and glycerolipid metabolism, both of which are critical for adipogenesis [49, 50]. The lncRNAs were divided into eight modules by expression level cluster analysis. Each module was associated with the four adipogenesis stages based on phenotypic association analysis. Four hundred forty-four lncRNAs were selected as candidate lncRNAs by restricting the module membership and gene significance. This research focused on the antisense DELs for the following reasons: 1. The reliability of candidate lncRNA prediction could be increased by integrating co-expression network analysis with differential analysis, as in other studies [51], which produce evidence from expression characteristics and sequence features. 2. The function of antisense lncRNAs can be predicted based on the sense strand gene, which is dependent on particular mechanism of the antisense lncRNAs [52]. Subsequent GO and KEGG analyses revealed that the target gene of *BIANCR* was involved in intramuscular adipocyte proliferation, apoptosis, and adipogenesis. More interestingly, the sense strand gene *MMP2* of *BIANCR* has indeed been proven to affect adipogenesis [53–55]. Furthermore, research has shown that more adipocytes are formed during the fetal and early postnatal stages than during the adult stages, which means that factors that positively/negatively regulate adipocyte differentiation may be more highly/lower expressed during the fetal stage [48], seven tissues (heart, liver, spleen, lung, kidney, subcutaneous fat, and *longissimus dorsi* muscle) from newborn (*n* = 3) calves and 48-month-old cattle (*n* = 3) were collected in this study to evaluate the role of *BIANCR* in bovine intramuscular adipogenesis. The *BIANCR* expression levels declined in fat and increased in muscle with aging, indicating that the expression levels of *BIANCR* could be a possible regulator that follows normal development principles of adipose tissues [48], but that *BIANCR* may play a different role in muscle and IMF. Compared with other tissues, *BIANCR* is highly expressed in adipose tissues (3^rd^) and longissimus dorsi muscle (1^st^) of newborn calves, and its expression level is significantly increased in the early stage (3 d) of intramuscular adipocyte differentiation. Moreover, *BIANCR* was located in the nucleus and cytoplasm in a uniform distribution, which means it possibly participates in gene transcription regulation and the ceRNA network [56, 57]. The above results were suggested that *BIANCR* played a key role in intramuscular adipogenesis.

Finally, the knockdown of *BIANCR* inhibited proliferation and adipogenesis and promoted apoptosis of the intramuscular adipocytes based on a functional loss experiment. Subsequently, the phosphorylation level of crucial proteins in the signaling pathway indicated that the inhibition of adipogenesis with the *BIANCR* knockdown was achieved through the ERK1/2 signaling pathway. The selection of signaling pathways was based on previous research and the KEGG analysis results of *BIANCR* [58–61]. ERK1/2 are members of the MAPK family [62], and the role of ERK1/2 in adipogenesis is complicated. On one hand, activation of ERK1/2 is required for adipogenesis and *PPARG* transcription; on the other hand, maintaining ERK1/2 dephosphorylation is required for *PPARG* expression during lipogenesis [63]. Based on this intricacy, this study clarified that inhibiting ERK1/2 signaling can increase *PPARG* expression and promote lipogenesis in bovine intramuscular adipocytes. During adipogenesis, the knocking down of *BIANC*R increased ERK1/2 phosphorylation and decreased *PPARG* expression levels; however, inhibiting ERK1/2 signaling reversed the effects. This result indicates that *BIANCR* enhanced adipogenesis by inhibiting the activation of the ERK1/2 signaling pathway. Notably, the inhibition of ERK1/2 signaling alone did not lead to a significant increase in Oil Red O-stained lipid droplets, which was inconsistent with the triacylglycerol assay. At 6 d and 9 d of lipogenesis, the situation was also observed in the Oil Red O staining and triacylglycerol assay. We theorize that after 6 d of differentiation, lipid accumulation in the intramuscular adipocytes reached a plateau. The variation in Oil Red O staining is insignificant for the limited lipid accumulation, but this cannot explain the increase in triacylglycerol levels, so more research is needed. Normally, regulators perform diverse roles during proliferation and differentiation to balance cell development. However, the findings of this study revealed that *BIANCR* positively regulated the proliferation and adipogenesis of intramuscular adipocyte, and this phenomenon has been observed in other lncRNA studies. Mouse-derived *lnc-ORA* have been shown to enhance adipocyte proliferation and differentiation simultaneously [64], while lncRNA *IGF2 AS* has been shown to stimulate proliferation and differentiation of bovine myoblast at the same time [65]. Therefore, we speculate that this particular phenomenon is specific to lncRNAs.

## Conclusions

In conclusion, this research presents a large number of lncRNA resources and a series of candidate lncRNAs for further investigation. We discovered and confirmed that the knockdown of *BIANCR* inhibited intramuscular adipogenesis through the ERK1/2 signaling pathway. This research is significant for improving beef meat quality, beef cattle directional selection, molecular breeding, and morbid obesity metabolic disease research.

## Supplementary Information

**Additional file 1.** Reads information of RNA-seq.

**Additional file 2.** Primers used in PCR and qRT-PCR.

**Additional file 3.** Antibody information.

**Additional file 4.** Sequencing data validated by qRT-PCR.

**Additional file 5.** Correlation analysis between samples.

**Additional file 6.** Type information of lncRNA.

**Additional file 7.** DELs between 4 adipogenesis stages.

**Additional file 8.** GO terms and KEGG pathways for DELs.

**Additional file 9.** Module information of WGCNA.

**Additional file 10.** Correlation between mRNA and BIANCR.

**Additional file 11.** GO terms and KEGG pathways for BIANCR.

**Additional file 12.** The sequence of BIANCR.

## Data Availability

The sequencing data have been submitted to the Gene Expression Omnibus (GEO) database (accession number: GSE185850).

## Abbreviations

ANOVA: Analysis of variance
*BIANCR*: Bovine intramuscular adipogenesis associated long non-coding RNA
ceRNA: Competing endogenous RNA
CNCI: Coding-noncoding index
CPC: Coding potential calculator
DELs: Differentially expressed lncRNAs
FCM: Flow cytometry
FPKM: Fragments per kilobase of transcript per million reads mapped
GO: Gene Ontology
IBMX: 3-Isobutyl-1-methylxanthine
IMF: Intramuscular fat
KEGG: Kyoto Encyclopedia of Genes and Genome
lncRNA: Long non-coding RNA
NC: Smart Silencer negative control
PBS: Phosphate buffer
PUFA: Polyunsaturated fatty acids
SD: Standard deviation
SS: Smart Silencer *BIANCR*
TAG: Triacylglycerol
WGCNA: Weighted gene co-expression network analysis
